# A qualitative study on incentive failure for college students in volunteer teaching programs

**DOI:** 10.3389/fpsyg.2026.1805438

**Published:** 2026-05-29

**Authors:** Li Li Liu, Tian Tian Shi

**Affiliations:** School of Foreign Languages, Harbin University of Science and Technology, Harbin, China

**Keywords:** grounded theory, incentive failure, organizational vitality, self-worth identity, volunteer teachers

## Abstract

**Introduction:**

Volunteer teachers are an important supplementary force for the development of education in underdeveloped areas at present. Nevertheless, problems such as weak retention intention, low work enthusiasm and high turnover rate among volunteers cannot be ignored. It is important to examine how to effectively incentivize volunteers to participate in volunteer teaching.

**Methods:**

This study focuses on the issue of incentive failure among volunteer teachers. From the perspective of qualitative research and based on the grounded theory methodology, the research team conducted in-depth interviews with 36 volunteer teachers and carried out participatory observation of the daily work of 11 volunteer teachers. Through constant comparative analysis of the data and repeated review of analytic memos, categories and subcategories were clarified using the three-stage coding process of open coding, axial coding, and selective coding, and a model of the mechanism of incentive failure among volunteer teachers was constructed.

**Results:**

The findings indicate that the extrinsic motivating factors leading to incentive failure among volunteer teachers include insufficient organizational vitality, deficiencies in the institutional framework, and imbalance in societal attention, while the intrinsic motivating factor is difficulty in self-worth identity. This study constructs a mechanism model of incentive failure for volunteer teachers.

**Discussion:**

Based on the above research findings, this study discusses strategies to improve the volunteer incentive mechanism and puts forward suggestions from the perspectives of organizational vitality, institutional regulation, social public opinion and school education.

## Introduction

Volunteers are an important force to make up for the shortage of teachers and promote educational equity (Jackson and Adarlo, 2016; Ackermann, 2019; Carlsen et al., 2022). Even in harsh refugee camps, there are volunteers who teach children with care (Baillie Smith et al., 2022; Meijeren et al., 2024). Evidence suggests that volunteer teaching is a volunteer program that volunteers enjoy participating in Villace-Molinero et al. (2024). Go West, a volunteer program for college students to support the western rural areas in China, which began in 1998, has recruited more than 30,000 volunteers to teach in over 700 county and township primary and secondary schools by 2024, with over 200 universities participating (China Youth, 2025). The program has made significant contributions to supplementing teacher resources in underdeveloped areas and promoting local education development (Luo, 2019). The study suggests that volunteer teachers are different from other types of volunteers, such as medical volunteers and sports event volunteers. Their recipients are mainly minors who expect to grow in terms of knowledge, moral character and psychology. This desire for growth makes the interaction between the recipients and the volunteers have distinct emotional characteristics. Volunteer incentive strategies should focus on this trait.

However, multiple studies have shown a decline in the number of people engaged in volunteer work in recent years (Kennedy and Brunold, 2015, p. 67; Thompson, 2022), has also a relatively high attrition rate among educational volunteer organizations (Garner and Garner, 2011; Smith, 2017), makes retaining volunteers an urgent problem to be addressed (Huynh et al., 2014). In China, college student volunteer initiatives operate within a government-guided framework, and participation is often subject to institutional encouragement (Wang, 2023). Research has shown an upward trend in the willingness of college students to volunteer (Mei et al., 2016; Zhao et al., 2018). Nevertheless, evidence also suggests that top-down incentives driven by the government are insufficient. A large-scale survey conducted by Zhang and Chen (2021) found that although 44.3% of college student volunteers received incentives, neither the frequency nor the duration of their participation showed a significant increase. As a result, how to incentivize volunteers became a research hotspot (Wilson, 2012; Studer and von Schnurbein, 2013; Hollas et al., 2022). Against this backdrop, this study focuses on the issue of incentive failure among volunteer teachers, employs qualitative research to reveal the complex mechanisms underlying such failure, and discusses the feasibility of several incentive strategies.

## Literature review

Existing studies on volunteer incentive have conducted in-depth analyses from aspects such as motive structure, incentive dimension, role identification, and organizational management.

### Motive structure

From the perspective of social system composition, volunteer activity has become an important part of the third sector due to its altruistic motives, task diversity, and complex relationships with professionals (Cnaan and Amrofell, 1994). In functional theory, Clary et al. (1998) and Clary and Snyder (1999) proposed that volunteering activities provide an opportunity to meet the diverse needs of individuals. Clary et al. (1998) believe that the six main motives that motivate people to engage in voluntary activities are: values, understanding, career, social, enhancement, and protection. When individuals engage in volunteer activities, they will seek different goals. In other words, different people have different motives although engaging in the same volunteer activities (Clary and Snyder, 1999). In recent years, more and more qualitative and quantitative studies have shown that volunteers participating in volunteer activities are driven by complex motives (Kiviniemi et al., 2002; Peloza et al., 2009; Stukas et al., 2016). This shows that the motives of volunteer activities are related to multiple functions, such as shaping values, improving understanding, protective function, social function, professional function (Rodell et al., 2016; Stukas et al., 2016), pro-sociality, interpersonal communication and learning opportunities, etc. (Hurst et al., 2019; Meijeren et al., 2024). Happiness and the realization of social value are also important motives for volunteers (Willems et al., 2012). The acceptance and appreciation of the environment affect the individual in a motivational sense in volunteering and continuing to volunteer, and students especially decide to volunteer due to the activities carried out in civil society organizations and universities (Aslan and Tuncay, 2023). In addition to the above motives, there are other psychological factors that have a certain impact on volunteers’ willingness to participate, including psychological pressure and psychological ownership (Ainsworth, 2020). Moreover, volunteer motives are constantly changing (O’Toole and Grey, 2015; Meyer and Simsa, 2018). It is obvious that volunteer motives are characterized by diversion, dynamism, and individuation and the research on volunteer motives is being continuously deepened and expanded.

### Incentive dimension

Research on incentive is often based on motive research. Volunteers’ personal tendencies and sociodemographic characteristics have been frequently studied. In the early 21st century, there have been many researches on volunteer motives and characteristics (Cnaan and Cascio, 1999; Bussell and Forbes, 2002; Musick and Wilson, 2008, p. 186; Hustinx et al., 2010; Rochester et al., 2010). Many studies suggest volunteer motives are important to match motive with incentive (Faletehan et al., 2021). However, only a few studies focus on the effects of appropriate matching strategies. The related studies focus more on the practice. Recognition, training, task assignment, volunteer development assistant are effective strategies to incentivize volunteers (Hager and Brudney, 2004, p. 121; Rende et al., 2023). A study tested the role of volunteer management practices in incentivizing rugby club volunteers (Cuskelly et al., 2006). It has made a “volunteer management checklist”, which includes items such as evaluation, recruitment, screening, induction training, support, performance management, and recognition. Another study also pointed out that training, continuous support and task matching have a positive motivation on the formation of volunteers’ identity and personal growth (Tang et al., 2010). Recent research has focused on how voluntary organization leadership incentivizes volunteers. For example, there has been a research that focused on four kinds of leadership, including self-supporting, structured, controlling and chaotic leadership. It is believed that self-supporting leadership and structured leadership are motivational while controlling leadership and chaotic leadership are amotivational.

### Role identification

Role identification is not only the internalized norms, but also a cognitive schema that provides a sense of meaning and shapes the impact on events and decisions (Stryker and Burke, 2000; Park, 2024). From the 1990s, role identification has attracted researchers’ attention. There is a significant correlation between low-level role ambiguity and organizational commitment (Nelson et al., 1995). The relationship between volunteer behavior and individual motives, role identification, and prosocial personality traits (Finkelstein et al., 2005). As participation continues, volunteer roles will be internalized and adopted as part of the self (Piliavin et al., 2002; Nichol et al., 2024). There is a significant correlation between identity and the time spent in volunteer activity (Finkelstein et al., 2005). It suggested that volunteers’ role identification should be cultivated by issuing t-shirts, license plate holder certificates and other items to them, so that their contributions can be recognized by the public. In addition, a study found that role recognition, role flexibility, role specification and integration are positive factors that affect volunteer role identification (Hong et al., 2009). Participating in a volunteer group can provide volunteers with a basis for defining their identity, which prompts researchers to make research on how to cultivate a sense of identity through the process of socialization and integration. A meaningful and purposeful life can regulate the impact of identity on the mental and physical health of volunteers, because the more time invested, the stronger the sense of identity and the more people can perceive their importance to others, which in turn enhances purpose and meaning (Thoits, 2012). Volunteers with low role identification and low perception of social support, high role identification and low perception of social support, low role identification and high perception social support can engage in volunteer activities faster when they have higher psychological capital (Xu et al., 2020). While, volunteers with high role identification and high-perception social support are more likely to engage in volunteer activities when they have lower psychological capital.

### Intention to remain

From the 1990s, there have been many explanatory models of volunteers’ intention to remain. Most of the explanatory variables contained in these models are individual factors (Chacón et al., 2007; Finkelstein et al., 2005; Omoto and Snyder, 1995), only a few variables are organizational variables, such as integration into the organization, satisfaction with organizational management, organizational commitment, etc. (Alfes et al., 2015; He et al., 2024). Organizational variables significantly affect employees’ remaining rate and intention (Yahaya and Ebrahim, 2016). Researches on the sustainability of volunteer activity show that when leaders are considered active, volunteers are more likely to stay in the organization for a long time (Catano et al., 2001; Avolio et al., 2004; Richardson and Vandenberg, 2005; Rowold and Rohmann, 2009; Senses-Ozyurt and Villicana-Reyna, 2016), and the leaders of voluntary organizations attract volunteers to stay in the organization through effective management practices and shaping the professional qualities of volunteers (Umezurike, 2011; Senses-Ozyurt and Villicana-Reyna, 2016; Benevene et al., 2018). Increasing volunteer participation will increase volunteers’ satisfaction with the organization and their intention to remain (Alfes et al., 2016; Vecina et al., 2012; Washinko, 2022). Aslan and Tuncay (2024) find that volunteer organization managers can effectively cultivate potential volunteers by establishing broad strategic partnerships with various organizations, universities, and other educational institutions.

Volunteer activity is a long-term planned behavior and is always in a process of dynamic change. Volunteers’ participation motives, role identification, and loyalty to the organization are affected by many factors (Omoto and Snyder, 1995; Penner, 2004). The literature review shows how the results of volunteer activities research from motive structure, incentive dimension, role identification, and intention to remain. However, due to the particularity of the recipients of volunteer teaching assistance, it is undeniable that there are still many areas to be explored. In addition to material support, what other factors affect the willingness of volunteers to participate? What are the mechanisms of interaction among these influencing factors? Accordingly, what adjustments should be made to the incentive strategy to achieve the desired effect? This study hopes to make useful explorations in these areas.

## Research design

This study takes college student volunteer teachers as the research subjects, adhering to the grounded theory paradigm, aiming to examine the causes of incentive failure among volunteer teachers and to further explore effective strategies for optimizing the incentive mechanism for volunteer teachers.

### Research methods and tools

Grounded theory emphasizes exploratory inquiry, the natural presentation of data, and rigorous research procedures, while also requiring researchers to maintain theoretical sensitivity at all times (Glaser and Strauss, 1967). The research team collaborated and immersed themselves in the context of volunteer teaching activities, conducting in-depth interviews and participatory observation, analyzing data through constant comparative analysis and memo-writing, and clarifying categories and subcategories through the three-stage coding process of open coding, axial coding, and selective coding. To ensure that the coding reflected the data as fully as possible, the team conducted multiple rounds of brainstorming, continuously comparing events, concepts, and structural relationships. After preliminary analysis, several rounds of theoretical sampling were carried out to develop and integrate categories. Meanwhile, NVivo 15 software was used to assist in data management and retrieval.

### Participants

In the initial phase of the study, the research team employed open sampling by selecting participants who could provide the maximum coverage of the research questions for in-depth interviews. In the intermediate phase, as concepts and categories gradually emerged, the team adopted relational and variational sampling, recruiting volunteers actively engaged in teaching support activities as the subjects of participant observation. The research team first selected a college student with 16 months of volunteer teaching experience for an unstructured interview. Through analysis of the interview data, six important concepts were identified: willingness, value, lack of experience, material deprivation, discrepancy between willingness and reality, and high morale. Based on these findings, the sampling criteria for the research subjects were established: (1) having more than six months of continuous or regularly intermittent volunteer teaching experience; and (2) being familiar with the basic procedures of recruitment, management, and evaluation of volunteer teachers. A total of 70 eligible volunteer teachers voluntarily participated in the interviews. Following the principle of maximum coverage of the research question in open sampling, the research team ranked the 70 volunteers according to the duration of their teaching service in descending order and selected those with more than 12 months of experience for interview data collection. Ultimately, 36 participants were included, forming 36 interview transcripts (coded ZA1–ZA36). Among them, 15 were female and 21 were male; 10 were aged 18–22 and 26 were aged 22–30; 10 were undergraduates and 26 were graduate students; 4 were from Willoong Voluntary Service, 6 from the School Youth Volunteer Association in college, and 26 from China’s Relay Program for Youth Volunteers Alleviating Poverty. Prior to the interviews, informed consent forms were signed with all participants to ensure voluntary participation. The research team also committed to maintaining the confidentiality of participants’ personal information, and all data were used solely for academic research. After the completion of the study, the handling of relevant data was conducted in accordance with the participants’ wishes.

Following an in-depth analysis of the interview data, concepts and categories gradually became clearer. The research team further employed relational and variational sampling methods to select volunteers who were actively engaged in teaching support activities for participant observation. The observation samples were mainly drawn from two locations: Chenggu County in Shaanxi Province, which served as a volunteer teaching site for a college in Northwest China, and Changwu County in Shaanxi Province, which served as a volunteer teaching site for a college in Northeast China. Upon arrival at the site, the research team contacted the volunteers and the four schools where the volunteers worked. Ethical approval was obtained from the schools’ ethics committees, and informed consent was secured from volunteers. In addition, the research team, through the schools, explained the purpose of the study to the students’ parents and assured them that the observation would focus solely on the teaching support volunteers and that no personal information of the local students would be disclosed. A total of 11 volunteer teachers (coded ZA37–ZA47) were observed, including 7 females and 4 males. All of them were graduate students, and their universities permitted them to suspend their studies during the volunteer teaching period. At the time the research team entered the field, these volunteers had already been working locally for seven months.

### Research process

After determining the research objectives, the research team conducted in-depth interviews, participatory observation, and coding, while memo-writing, diagram construction, and reflection ran throughout the entire research process.

## Data collection

This study developed a semi-structured interview protocol aligned with the research objectives. The protocol comprised two sections: the first collected participants’ demographic information, while the second focused on volunteers’ reflections on their teaching experiences, evaluations of the program, and suggestions for improvement. For example, “Do you think volunteer teaching is meaningful? Why?” “What benefits do you think your volunteer teaching brings to the recipient areas?” “What motivates you to persist in volunteer teaching?” “What experiences during volunteer teaching have left a deep impression on you?” and so on. Data collection primarily involved face-to-face interviews, supplemented by online video sessions when necessary. Each interviewee’s interview is limited to 30 to 50 min. Through preliminary organization of the interview data, the research team identified several emerging elements, such as recipient areas’ skepticism toward college student volunteers, difficulties in communication between volunteers and parents, and interactions with local children. With these emerging insights, the research team entered the context of college student volunteer teaching activities.

The research team conducted a 20-day participatory observation survey in Chenggu County and Changwu County, Shaanxi Province, observing the daily work of 11 volunteers, including classroom teaching, after-school tutoring, speech contests, home visits, and guiding students in club activities. During the research period there, nearly 30,000 words of observation notes were written and a large number of photo materials were taken.

## Memo-writing

Memos not only record the researcher’s questions and analyses but also present the researcher’s thinking trajectory and reflections, serving as a stage where facts and ideas collide (Glaser and Strauss, 1967). The research team wrote memos for each interview transcript, each day’s participant observation, and each team discussion. These memos were broadly categorized into interview/observation memos, coding memos, and theoretical memos, resulting in a total of 56 memos comprising nearly 40,000 Chinese characters. The constant comparative analysis—including coding, theoretical sampling, clarifying categories, and developing concepts—was conducted based on both interview data and memos. Below is an example of a theoretical memo, which demonstrates the positive role of researcher reflection in the formation of theory. Title: Where Does the Motivation for Teaching Support Come From?

*Although volunteers recounted numerous difficulties—such as lack of drinking water and no indoor heating—they nevertheless persisted in their teaching support activities. What reasons prevented them from discontinuing their volunteer teaching? Beyond utilitarian motives such as skill development, obtaining honors, and accumulating social capital, they also mentioned the growth of the children, the recognition of parents, and altruistic motives as significant facilitators of their teaching support behavior.*

## Coding

The research team conducted coding of the interview data and observation logs using open coding, axial coding, and selective coding. The coding process was completed independently by two researchers. Prior to formal coding, the coders conducted coding tests and calibration to ensure the objectivity of the conceptualization process. After the initial concepts were formed, the two coders engaged in thorough discussions to delete or integrate contradictory or inconsistent initial concepts. The extraction of sub-categories and main categories emerged through an iterative process of constant comparative analysis involving extraction, comparison, induction, refinement, testing, revision, and confirmation. First, the textual data were coded line by line with original concept labels assigned, followed by integration and categorization, ultimately resulting in 53 initial concepts (a1–a53) and 18 subcategories (A1–A18). Table 1 presents an example of the conceptualization process of original statements, and Table 2 illustrates the process of deriving subcategories from initial categories.

**Table 1:** Open coding (example).

Category	Conceptualization of original statements	Initial concepts
Interview data ZA5	Z1 participated in a volunteer teaching activity at a primary school as a caregiver (primary school caregiving); Z2 from registration to on-site participation took a three-month preparation period (three months); Z3 attended multiple preparatory meetings and training sessions, with several changes in meeting locations during the process (location changes); Z4 had to become familiar with everything again each time, which was quite frustrating (irritation).	a6. Preparation duration; a7. Frequent changes of location; a12. Caregiving
Observation Data ZA40	Z1501 the volunteer served as a Chinese teacher and head teacher of the class (head teacher); Z1502 used a microphone in class and presented content with PPT courseware, using physical teaching aids such as seeds (use of teaching aids); Z1503 asked students to come to the front and plant seeds in flowerpots (planting); Z1504 students were unusually excited, some listened attentively while others played at the back of the classroom (strong response); Z1505 after teaching, students were asked to complete test papers (test); Z1506 prepared for final examinations, and students reported having a short test in every class (final exam preparation).	a19. Teaching methods; a22. Final performance

Z1 ~ indicates the serial number of sentence-by-sentence coding.

**Table 2:** Results of subcategory derivation.

Initial concepts	Subcategory	Initial concepts	Subcategory
a1. Grassroots organizations a2. Project approval	a1. Organizational support	a27. Eligibility for postgraduate recommendation a28. Perseverance development a29. Serving hometown a30. Public welfare sentiment	a10. Diversity of motives
a3. Activity expenses a4. Transportation expenses a5. Personal financial burden	a2. Financial support	a31. Omission of names a32. Back seating position	a11. Integration into team
a6. Preparation duration a7. Frequent changes of location	a3. Spatial support	a33. Newcomer status a34. Student transfer a35. Loose classroom discipline	a12. Expectation alignment
a8. Learn-from-Lei-Feng day a9. Labor day a10. Spring festival a11. First day of school	a4. Campaign-style projects	a36. Heating a37. Sun protection a38. Lack of furniture a39. Lack of kitchen a40. Medical insurance	a13. Living materials
a12. Caregiving a13. Recreational activities a14. Extracurricular activities a15. Safety-oriented	a5. Recreational projects	a41. “Résumé-building”; a42. Lack of experience	a14. Local acceptance
a16. Participation in competitions a17. Attending exchange meetings a18. Award speeches	a6. Evaluation climate	a43 form-based evaluation a44 self-submitted evaluation a45 praise-Oriented evaluation	a15 Self-evaluation dominance
a19. Teaching methods a20. Student conflicts a21. Student injuries a22. Final performance	a7. Classroom management ability	a46. Observation-based evaluation a47. Verbal praise a48 avoidance of problems	a16. Formalized evaluation
a23. Excessive meetings a24. Educational poverty alleviation tasks a25. Rural visits a26. Excessive documentation	a8. Administrative work	a49. Praise-oriented a50. Supplicatory attitude a51. Neglect of mistakes	a17. Praise-dominated evaluation
a32. Dusty environment a33. Poor sanitation a36. Rudimentary accommodation	a9. Environmental adaptation	a51. Large-scale projects a52. Major events a53. Self-media promotion	a18. Grand narrative

The research team, through repeated review of analytic memos, continuous comparison of events, and analysis of contexts and relationships, identified eight major categories (B1-B8) on the basis of open coding: a limited number of volunteer teaching organizations, hollow project content, insufficient training, unstable willingness to engage in volunteer teaching, misalignment of value identification, insufficient material support, lack of evaluation mechanisms, and insufficient public communication, as shown in Table 3.

**Table 3:** Axial coding and derivation of major categories.

Major category	Subcategory	Category connotation
B1. Limited number of volunteer teaching organizations	A1. Organizational support	Non-governmental volunteer organizations must rely on affiliated institutions
	A2. Financial support	Lack of stable funding sources
	A3. Spatial support	Lack of stable office space and personnel
B2. Hollow project content	A4. Campaign-style projects	A large number of “campaign-style” activities during holidays
	A5. Recreational projects	Short-term projects are dominated by recreational activities
	A6. Evaluation climate	Various forms of evaluation weaken the sense of meaning in volunteer teaching
B3. Insufficient training	A7. Classroom management ability	Lack of knowledge in teaching methods, classroom management, and students’ mental health
	A8. Administrative work	Lack of understanding of non-teaching tasks such as meetings and report writing
B4. Unstable willingness to volunteer teach	A9. Environmental adaptation	Adaptation to harsh environments requires prior training
	A10. Diversity of Motives	Diverse motivations for participation, such as further study or personal sentiment
B5. Misalignment of value identification	A11. Integration into team	Difficulty integrating into local teacher teams, leading to a sense of being an “outsider”
	A12. Expectation alignment	Parents’ lack of confidence in volunteers serving as head teachers
B6. Insufficient material support	A13. Living materials	Inadequate provision of daily necessities for long-term volunteer teachers
	A14. Local acceptance	Lack of perceived support and enthusiasm from local residents
B7. Lack of evaluation mechanisms	A15. Self-evaluation dominance	Evaluations initiated by official organizations are mainly based on volunteers’ self-evaluation
	A16. Formalized evaluation	Evaluations by non-governmental organizations are mainly conducted through superficial visits
	A17. Praise-dominated evaluation	Evaluations in recipient areas are mainly based on praise
B8. Weak public communication	A18. Grand narrative	Social media mainly focuses on large-scale projects and major events

Continuous comparison and systematic analysis of the major categories in axial coding led to the identification of the core category as the analysis of factors contributing to incentive failure among college student volunteer teachers. Through multiple rounds of brainstorming and theoretical sampling, the research team continuously analyzed the eight major categories, grouping B1, B2, and B3 under organizational vitality; B4 and B5 under self-worth identity; B6 and B7 under institutional framework; and B8 under societal attention. The research team conducted theoretical sampling of the major category B8, repeatedly reviewing the data and analytic memos to examine the possibility of further developing and enriching this category. Based on the existing data, no additional dimensions were identified. At this point, the “storyline” of this study has become clearly apparent: insufficient organizational vitality reduces volunteers’ level of professionalism; inadequate management systems weaken volunteers’ sense of security; difficulties in self-worth identity hinder volunteers from forming a firm sense of meaning; and imbalanced social concern affects public perception. These factors collectively interact to impede the positive functioning of incentive mechanisms for teaching support volunteers.

## Model construction

**Figure 1:**
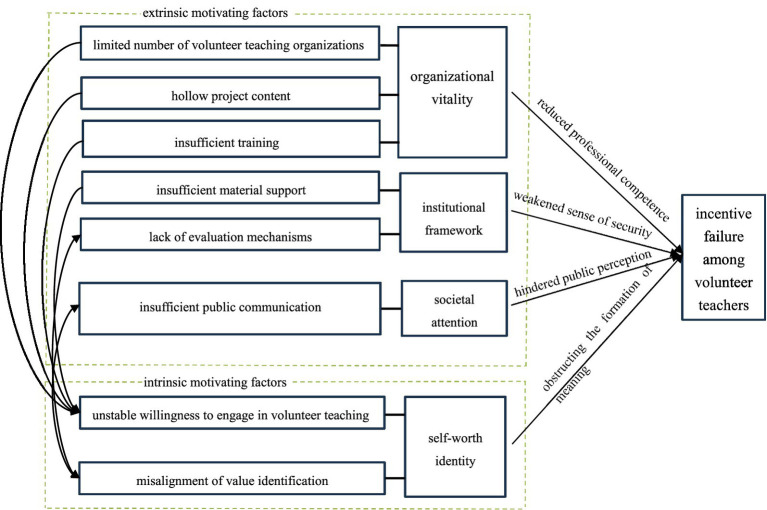
A mechanism model of incentive failure in volunteer teaching programs.

Based on this “storyline,” this study constructed a model of the mechanism of incentive failure among college student volunteer teachers, as shown in Figure 1. The extrinsic motivating factors leading to incentive failure among volunteer teachers include organizational vitality, institutional framework, and societal attention, while the intrinsic motivating factor is difficulty in self-worth identity. There is an interaction between extrinsic and intrinsic motivating factors: the limited number of volunteer teaching organizations, hollow project content, insufficient training, and inadequate material support weaken volunteers’ willingness to engage in volunteer teaching. The lack of evaluation mechanisms and insufficient public communication lead to a mismatch between public value identification and volunteers’ value identification; in turn, the decline in volunteers’ value identification toward volunteer teaching also affects the depth of evaluation and the intensity of media attention. Regarding the test of theoretical saturation, on the one hand, the research team comprehensively reviewed the collected data and analytic memos; on the other hand, five volunteers who met the recruitment criteria but were not selected as interview participants were randomly sampled and interviewed to test theoretical saturation. The results showed that no new concepts or dimensions emerged, thereby confirming that the theoretical model of this study reached saturation.

## Results

### Insufficient organizational vitality results in reduced professional competence

Volunteer organizations depend on the work of volunteers to fulfill their missions. However, these organizations often have limited financial or other resources to reward, motivate, and retain volunteers. Therefore, it is crucial to fully leverage means beyond material incentives to encourage volunteers’ intention to stay (Nesbit et al., 2016). This study shows that organizational vitality directly affects volunteer participation willingness. Deficient organizational vitality manifests specifically through: (a) limited number of volunteer teaching organizations, (b) hollow project content, (c) insufficient training. Volunteer teaching activities planned by non-governmental organizations have imposed a considerable burden on volunteers due to factors such as frequent changes of leaders and lack of activity funds (a3; a6; a7). Ninety-seven percent of participants said that the content of the project should first be beneficial to others, and secondly, the participants should be competent (a12; a13; a19). Tokenistic or ritualistic volunteer teaching programs demonstrably fail to inspire volunteer engagement or secure stable recruitment, revealing volunteer resistance to campaign-style activities (a8; a9; a10; a11). Volunteer teachers generally hope to receive training in teaching methods, classroom management, child psychology, and related areas (a20; a21; a22). Multiple studies have shown that measures such as skills training, creating a democratic work climate, and organizing seminars to facilitate exchanges between paid staff and volunteers can significantly enhance volunteers’ professionalism and satisfy their competence needs (Hager and Brudney, 2004, p. 203; Rogelberg et al., 2010). The frequency, depth, and effectiveness of implementing these measures are key indicators of whether a volunteer organization is vibrant (Löfström, 2025). The volunteer organizations to which the participants belonged stated the importance of skills training in their action protocols, but the implementation was uneven. Research by Jensen and Thomsen (2022) has shown that negative stereotypes and distant attitudes held by paid staff toward volunteers may lead volunteers to feel unwelcome, unaccepted, and unappreciated, due to the questioning of volunteers’ skills by both paid staff and service recipients. It is evident that volunteers’ level of professionalism has a significant impact on both their ability and willingness to remain in their roles.

### Deficiencies in the institutional framework undermine the sense of security

The role of volunteer organizations and other non-governmental organizations in public policy has been described as a fundamental feature of modern government (Salamon, 1995). In numerous policy domains, policymakers increasingly rely on non-governmental organizations such as volunteer organizations to achieve their goals, as these organizations possess greater autonomy, innovativeness, and flexibility (Hall, 2000). In China, the institutional logic of volunteer organization development originates from the socialist system with Chinese characteristics (Zhao and Wu, 2020), within which the Party’s leadership and a unified political framework provide comprehensive policy guidance—covering the legal system, macro-level planning, and extending to decentralization and the procurement of public services (Yuen, 2020).

Most of the respondents in this study came from the China Graduate Volunteer Teaching Program for Poverty Alleviation, an organization that receives government funding and recruits and manages volunteers within the framework prescribed by the government. However, the prevalent issue of inadequate material support in recipient regions has significantly undermined volunteer motivation, especially for long-term volunteers serving over one year who rely on host communities for basic living provisions—a responsibility frequently neglected (a36; a37; a38; a39; a40; a41; a42). Chenggu County’s School A provided only basic tableware, School B added a 1,600 W heater, and School C supplied an induction cooker, rice cooker, and tableware, while Changwu County’s Schools D and E offered nothing beyond dormitories and beds. This systemic neglect stems from recipient areas perceiving volunteers as low-cost labor rather than skilled contributors, ultimately jeopardizing program sustainability. Even under such circumstances, multiple participants emphasized the significance of their interactions with aid recipients, particularly children, with 60% maintaining ongoing contact.

Evaluation is an important part of the organization’s management process. Fair and impartial evaluation enables volunteers to fully trust the organization’s judgment, meet their self-development needs, and make them willing to follow the organization (Devereux, 2008). The findings of this study show that there are common problems such as rigid evaluation methods and lack of process evaluation in both government-led volunteer projects and those initiated by civil volunteer organizations. As shown in Figure 1, the evaluation methods are either by excessive bureaucratization and tokenism, or by superficial inspections (a46; a47). Such evaluations not only fail to incentivize volunteers in any way, but also lead some volunteer organizations to attach too much importance to the external form and have a prominent utilitarian tendency (a48; a49). 92% of the respondents in this study came from government-funded volunteer organizations. Their involvement in activities such as material support, evaluation, and training for teaching support initiatives was largely dominated by government agencies, thereby squeezing the space for autonomous decision-making by volunteer organizations and volunteers to a certain extent. Therefore, policy interventions aimed at strengthening the sound operation of volunteer organizations must align with China’s institutional logic, cultural norms, resource endowments, and significantly varied regional governance conditions (Zhao et al., 2025).

### Imbalance in societal attention impairs public perception

In modern society, where information is transmitted at high speed, the media play a highly active role in society. The news system and society as a whole are essentially in a relationship of mutual influence and interaction, involving not only the dependence of news on society, politics, and the economy, but also the active role and impact of the news industry, news media, news communication, and news itself on the overall development of society and changes in its various subsystems (Freberg et al., 2011). The areas that receive media attention are more likely to become matters of societal attention. In particular, reports by mainstream media often play a role in shaping public perception (Neubaum and Krämer, 2017). Mainstream media in China bear political and ideological responsibilities and tend to focus on major social events, conducting reporting activities centered on government affairs (Huang et al., 2023). Reports on volunteer teaching are scattered across newspapers with relatively small audiences, such as China Youth Daily, as well as self-media platforms including WeChat official accounts and blogs established by volunteers (a51; a52; a53). The evident result is that volunteer teachers appear infrequently in the public eye, lack in-depth coverage, and present an underdeveloped public image. This leads the public to assume that the shortage of teachers in underdeveloped areas has probably been resolved, that volunteer teachers likely exist, that volunteers are entirely motivated by altruism, and that the issue is largely irrelevant to themselves, resulting in a widespread sense of false reassurance. In a social atmosphere where most people remain indifferent, volunteer teachers may begin to question their value identification: is volunteer teaching meaningful?

### Difficulties in self-worth identity affect the development of volunteers’ sense of meaning

According to self-determination theory, the potential for self-determination can guide individuals to engage in behaviors that are interesting and beneficial to their own competence development, and this pursuit of self-determination constitutes the intrinsic motivation for human behavior (Deci and Ryan, 2000). The initial motivation of volunteers to participate in volunteer activities is strongly influenced by the autonomy needs posited by self-determination theory; however, psychological mechanisms such as transcendent motivation and role identity are significant in strengthening sustained participation in volunteer activities (Ding et al., 2026). During the interviews, all 36 participants talked about the issue of self-worth without exception, which shows that the volunteer teachers have a very deep understanding of the teaching cause, and their motivation goes far beyond utilitarian factors and rises to the level of self-worth realization (a27; a28; a30). Challenging self-worth identity is the root cause of the incentive failure, mainly manifested in confusion of identity and lack of belongingness. Ideally, with increased duration and frequency of participation in volunteer teaching, volunteers’ sense of teacher identity and belongingness to the teacher community should progressively strengthen. By continuously participating in volunteer activities, volunteers internalize the volunteer role as part of their self-concept to recognize their self-worth, a process that benefits from clear and purposeful role planning (Bowe et al., 2020; Restler and Glant, 2020). Positive self-worth identity promotes optimal functioning, greater sense of efficacy, development (self-enhancement), well-being, life satisfaction, better performance, and task persistence (Donnellan et al., 2005). However, empirical data reveal a recurring dilemma wherein volunteer teachers frequently encounter the predicament of being perceived as outsiders (a31; a32). Both recipient schools and students’ guardians exhibit exclusionary attitudes towards volunteers (a33; a34; a35). As illustrated in Figure 1, When volunteer teachers are unable to affirm their teacher identity in a timely manner and fail to experience genuine acceptance from the local teaching community, the continuity of their volunteer teaching becomes severely compromised. Volunteers who experience prolonged periods of confusion regarding their sense of meaning face a risk of attrition: How do volunteers cope with the tension between their own expectations and the pressures of market logic? How do they cope with discrimination and distorted evaluations from paid staff?

## Discussion

Incentive is an inherently complex construct, deeply intertwined with the unique socio-cultural context of each nation (Park, 2024). This study attempts to explore strategies for optimizing the incentive mechanism for volunteer teachers from four dimensions: organizational vitality, institutional framework, societal attention, and public morality education.

### Fully activate the organization’s vitality

Organizational management serves as a critical safeguard for the effective implementation of volunteer teaching initiatives (Berkes, 2009). Relevant studies in the fields of organizational psychology and sociology indicate that social working conditions, interpersonal relationships, and organizational culture are positively correlated with employees’ work vitality (Carmeli et al., 2009; Macey and Schneider, 2008), among which organizational culture is regarded as an important supporting factor in satisfying basic psychological needs (Pelletier et al., 2004). As a concrete manifestation of organizational culture, leadership style and working style are frequently incorporated into research themes. Although volunteer organizations differ from paid work organizations, their organizational structures share certain similarities, and both organizational structure and managerial style are key factors in stimulating volunteer vitality (van Scheppingen et al., 2015).

However, the current landscape of civil volunteer organizations in China reveals significant limitations, including a scarcity of organizations, limited project diversity, inadequate organizational management, insufficient volunteer training, and weak fundraising capabilities. These challenges largely stem from the immature development of grassroots volunteer organizations. Thus, fostering the rapid growth and professionalization of such organizations is imperative. And shifting internal management towards a cooperation-oriented model is essential. Although both modern volunteerism and traditional philanthropy share moral characteristics of public welfare (Salamon, 1987; De Clerck et al., 2021), their operational paradigms diverge significantly. In volunteer activities, roles between volunteers and recipients are fluid, volunteer groups form organically, and partnerships are established based on shared aspirations (Salamon and Anheier, 1992; Chang et al., 2012). Cooperation embodies equality in status and discourse, necessitating a transition from control-oriented to collaboration-oriented management strategies. A paradigm shift in organizational philosophy is therefore pivotal. Organizations must leverage the inherent appeal of volunteer projects, cultivate a culture of unity and cooperation, and provide platforms for volunteer interaction and mutual learning. Through this process, organizational values can be internalized as the moral will of volunteers, thereby fostering a heightened sense of moral responsibility and intrinsic discipline. Consequently, volunteers’ cooperative awareness, abilities, and skills can be continuously strengthened through practice.

An increasing number of studies indicate that leadership in volunteer organizations is a key factor influencing volunteers’ satisfaction and commitment, thereby affecting their turnover, intention to remain, performance, and well-being (Senses-Ozyurt and Villicana-Reyna, 2016; Yahaya and Ebrahim, 2016). Transformational leadership, servant leadership, empowering leadership, and leadership that promotes volunteer learning and innovation, among others, are all positively correlated with volunteers’ intention to remain (Schneider and George, 2011; Greenleaf, 2019; Benevene et al., 2020), whereas transactional leadership and laissez-faire leadership are negatively correlated with intention to remain (Aboramadan and Kundi, 2020). According to self-determination theory, when leaders provide sufficient autonomy support, positive outcomes can be expected, as followers’ three basic psychological needs—autonomy, competence, and relatedness—are satisfied (Deci and Ryan, 2000). Volunteers invest their time and energy in organizations without obligation or financial compensation; therefore, leaders should provide volunteers with choices, encourage and actively seek their participation, opinions, and initiative, listen to their personal preferences, interests, and aspirations, and empathize with the negative emotions they express (Ryan and Deci, 2017). In general, whether transformational leadership or autonomy-supportive leadership is more effective in stimulating volunteer vitality should be determined on the basis of analyzing volunteer characteristics, adhering to a realistic approach, and taking into account individual and project-specific differences.

### Legalize and de-bureaucratize the institutional framework

The government’s management objective for civil volunteer organizations should be to establish a modern social organization system characterized by the separation of government and society, a clear delineation of rights and responsibilities, and self-governance in accordance with the law, thereby facilitating an organic interaction between the government and civil volunteer organizations in the provision of public services and the management of social public affairs (Salamon, 1995, p. 90). The realization of this objective depends on a sound legislative environment and a de-bureaucratized management approach. This study shows that material support and evaluation feedback are important components of the volunteer teaching environment and serve as extrinsic motivating factors for sustaining volunteer teachers’ participation in volunteer activities. Whether public or private volunteer organizations, their material fundraising, volunteer recruitment, and project development all require a legal basis (Barnes and Sharpe, 2009). Only a de-bureaucratized management approach can relieve volunteer organizations of administrative constraints, enabling managers to disengage from cumbersome, perfunctory, and superficial tasks and to devote their efforts to substantive and effective evaluation and feedback.

Enhancing the legislative framework is a foundational condition for supporting the growth of volunteer organizations. While landmark policies, such as the State Council of the People’s Republic of China (2017) and the People’s Republic of China National People’s Congress. (2016), have marked significant progress, the scope and depth of relevant legislation require further expansion and acceleration. Furthermore, relaxing the legal requirements for volunteer organizations to obtain legal person status remains an urgent task. Implementing a qualification-based registration review system, where the registration authority examines whether the organization has the qualification to register as a legal person, would serve as a pivotal entry point for advancing the legalization and professionalization of grassroots volunteer organizations. For instance, in February 2017, the Enterprise Income Tax Law of the People’s Republic of China significantly expanded tax benefits for corporate charitable donations—from 3 to 12% of annual profits (Huang, 2018, p. 201)—effectively stimulating corporate engagement in public welfare. Concurrently, it is imperative to develop private philanthropic capital and diversify funding channels.

The reality that China is facing is that the traditional centralized governance model is increasingly ill-suited to address the complexities arising from modern social transformations (Liu and Wei, 2018). Therefore, it is essential to transfer certain public functions to civil volunteer organizations and adopt a participatory, multi-stakeholder governance approach. This process requires a dual strategy: offering policy support while simultaneously establishing robust reward and penalty mechanisms to ensure dynamic organizational renewal. Effective practices include voluntary initiation, self-elected leadership, self-funding, self-managed personnel, and independent operations. Eliminating administrative rankings, official staffing quotas, supervisory departments, and part-time governmental appointments will further safeguard the independence and civic nature of volunteer organizations.

A well-functioning volunteer organization should be able to achieve the following two aspects. Firstly, volunteer organizations should proactively disclose operational information. Unlike corporations, volunteer organizations do not possess proprietary rights to confidential information (De Clerck et al., 2021). Transparent disclosure regarding organizational operations, project implementation, financial management, and administrative affairs is vital for public trust. Secondly, independent third-party evaluations are indispensable. Based on standardized evaluation metrics, third-party agencies should assess fund utilization, procedural compliance, and the relevance and appropriateness of services provided to beneficiaries (Deng, 2004), with findings disclosed promptly to the public. These agencies serve as vital guarantors bridging volunteer organizations and public resources.

### Develop diverse communication channels to attract societal attention

Wachinger et al. (2013) point out that individuals lacking personal experience rely more heavily on media narratives in risk assessment. Media significantly shape social risk perception (Paek and Hove, 2017). Contemporary society is characterized by the coexistence of traditional media—such as newspapers, television, and radio—and digital media represented by blogs, podcasts, and interactive wikis. Traditional media, with their rigorous modes of information dissemination, fulfill functions of environmental monitoring and social coordination (Niu et al., 2022), while digital media, by breaking the monopoly of traditional media over discourse, have rapidly emerged as new channels of information dissemination, characterized by ubiquity, personalization, and interactivity (Won et al., 2025). Therefore, this study argues that attracting societal attention to volunteer teaching initiatives requires the combined efforts of both traditional and digital media.

In situations where information is extremely abundant and opinions are highly complex, individuals often face decision-making difficulties, which in turn leads to a decline in social cohesion (Daume et al., 2023). Due to their operational models and the presence of gatekeepers, traditional media are able to identify the most important information from a mass of disordered information and phenomena, greatly reducing the time and effort required for individuals to understand the world. They are also adept at effectively linking different events, opinions, and claims, thereby compensating for the limitations and deficiencies of intuitive social cognition and enabling a more comprehensive grasp of reality, thus maintaining social cohesion (Boezeman and Ellemers, 2008). Therefore, volunteer teachers should strive to gain the attention of traditional media and maintain regular exposure in publications and broadcasts.

The term “new media” has not yet formed a definition that is widely accepted in academia since its emergence. In terms of current development, new media refer to emerging media that are based on digital technology, communication network technology, internet technology, and mobile communication technology, providing users with information, content, and services (Sundar and Limperos, 2013). Today, new media have even replaced the “opinion leader” role of traditional media (Zhang, 2025). Against the background of the widespread use of smartphones, volunteer teachers are not only participants in volunteer teaching activities but also narrators of these initiatives, and may even become effective operators of new media platforms. In response to audiences increasingly accustomed to “digital reading,” telling the “stories of volunteer teaching” requires not only content-oriented approaches but also the adoption of narrative modes, discursive strategies, and technological means suited to new media communication, enabling personalized, visualized, and interactive presentation. Efforts may focus on producing such narratives through “three micro and one client” platforms (microblog, WeChat, short videos, and news apps), and may also involve participation in various public digital service platforms embedded in communities and rural areas, such as smart city and smart village initiatives, in order to enhance audience engagement.

### Foster public morality education in everyday educational practices

Self-determination theory posits that the satisfaction of three basic psychological needs—autonomy, competence, and relatedness—is essential for individuals to achieve psychological growth, internalization, and well-being. Psychological growth is typically manifested through intrinsic motivation, that is, individuals’ curious and exploratory engagement in activities that are interesting and enjoyable, even in the absence of external reinforcement (Deci and Ryan, 2000). The psychological growth of volunteer teachers is reflected in the formation of values and their firm enactment, that is, identifying with the value of volunteer activities and recognizing their own value within such activities. Whether volunteers can achieve such psychological growth depends on whether they can obtain the necessary nourishment (den Van Broeck et al., 2016). Public morality education provides precisely the most suitable foundation for nurturing the formation of volunteers’ value systems.

The core objective of public morality education is to cultivate civic virtues, with a particular emphasis on fostering a sense of civic responsibility and a spirit of communal benefit (Banks, 2017). The spirit of benefiting the public is the root of the budding sense of volunteerism. Within the intertwined domains of family, school, and society, family members—especially parents—serve as primary “significant others,” exerting profound influence through their role-modeling behaviors (Restler and Glant, 2020). Empirical studies indicate that adolescents with strong civic consciousness often emerge from families with deeply ingrained traditions of altruism (Hoskins et al., 2011). In parallel, schools represent the principal arena for formal public morality education. Reform can be attempted from two dimensions: first, adopting democratic management of classroom affairs; second, establishing a public morality curriculum system that integrates theory and practice. Through democratic approaches, students can be guided to participate in formulating classroom rules. In the process of personally engaging in rule-making, on the one hand, students can develop a deeper understanding of the intrinsic value of rules and the difficulty of coordinating various interests, thereby increasing their sense of affinity toward rules; on the other hand, it can enhance their sense of collective ownership, effectively alleviating the negative resistance that may arise from the passive enforcement of rules (Castilla-Estévez, 2025). Regarding public morality education, every country incorporates such courses into its knowledge-based curriculum system, such as “Ideological and Moral Education,” “Morality and Society,” and “Morality and Life,” educating students through role models, exemplary figures, or counterexamples (Castilla-Estévez and Blázquez-Rincón, 2021). The importance of theoretical instruction is self-evident; however, practice enables adolescents to directly experience the power of sharing, coexisting, mutual respect, and collective responsibility.

Encouraging youths to independently explore avenues for moral action, exemplified by simple acts such as rescuing a stray dog, can foster more profound moral development than repetitive, ceremonial charitable activities. Humans inherently aspire toward truth, goodness, and beauty, an aspiration that is universally expected by society of its youth. In striving to meet these societal expectations and attain positive recognition, young people also develop an intrinsic desire for self-improvement and moral growth (Soong, 2013). Consequently, with proactive guidance from families, schools, and the media, public morality education can effectively take root in everyday educational life, thereby nurturing a robust sense of volunteerism.

## Conclusion

This study explores the issue of incentive failure among volunteer teachers from the perspective of qualitative research. The findings indicate that insufficient organizational vitality leads to low professional competence among volunteer teachers, making it difficult for them to develop a sense of competence in volunteer activities. Deficiencies in the institutional framework result in a lack of sense of security and reduced trust in organizations. Imbalance in societal attention leads to a mismatch between public value identification and that of volunteer teachers, hindering the widespread dissemination of volunteer values. Difficulty in self-worth identity constitutes the intrinsic motivating factor underlying incentive failure, as volunteer teachers remain caught in a prolonged search for role identification and a sense of belonging within volunteer activities, making it difficult to form firm commitment to volunteer teaching, and causing volunteer activities to gradually become uninteresting and unpleasant. Based on the above findings, this study proposes strategies for optimizing the incentive mechanism for volunteer teachers. The conclusions of this study contribute to improving the support conditions for volunteer teachers and, in turn, attracting more volunteers to engage in educational initiatives. The innovation of this study lies in revealing the mechanism of extrinsic and intrinsic factors within the volunteer incentive system. A well-established legal environment, supervisory and evaluative environment, and public opinion environment are important components of the volunteer action support system, and these factors profoundly influence the formation of volunteers’ self-worth identity. Compared with identified factors such as organizational identification, altruistic motivation, and empathy, self-identity has stronger explanatory power for the effectiveness of incentive mechanisms.

A limitation of this study lies in the uniqueness of China’s national context. College student volunteer activities in China are often institutionally encouraged, organized, and structured. Many volunteer projects are implemented by college administrative units or student organizations, or are integrated into social practice courses, with participation linked to academic credits or eligibility for awards and evaluations. For student volunteers participating in these structured projects, volunteer activities are not merely an expression of self-worth or altruism, but also a process of fulfilling a student role that carries implicit expectations. In this context, whether volunteers can successfully internalize a volunteer identity becomes crucial for sustaining long-term participation. On the other hand, this structured environment may also amplify the importance of identity-based pathways for persistent retention, while attenuating the influence of other factors—such as empathy—on motivating retention intentions. This represents one of the future directions for this study to explore. In addition, in recent years, grassroots volunteer organizations have achieved considerable development in China. Their operational status and volunteer motivation practices will also constitute a subject of this study.

## Data Availability

The original contributions presented in the study are included in the article/supplementary material, further inquiries can be directed to the corresponding author.
